# Supplementary material for: Nonintensive Care Unit Norepinephrine as an Alternative to Terlipressin in Hepatorenal Syndrome–Acute Kidney Injury
Source: Gastro Hep Adv. 2026 Aug 12;5(11):101096. doi: 10.1016/j.gastha.2026.101096 (PMC13584103; doi:10.1016/j.gastha.2026.101096)
Supplement: Supplementary file 1 [file mmc1.pdf]

**Supplementary Table 1. Baseline characteristics of patients that tolerated terlipressin versus discontinued**

| Characteristic | Tolerated (n=31) | Discontinued (n=19) | p-value |
|---|---|---|---|
| Median age, years (IQR) | 52 (42-58.5) | 60 (39.5-70) | 0.31 |
| Male sex, n (%) | 23 (74.2) | 15 (78.9) | 1.00 |
| Race/Ethnicity, n (%) | | | 0.74 |
| Hispanic/Latino | 14 (45.2) | 7 (36.8) | |
| Non-Hispanic White | 11 (35.5) | 8 (42.1) | |
| Non-Hispanic Black | 0 (0.0) | 1 (5.3) | |
| Non-Hispanic Asian | 3 (9.7) | 2 (10.5) | |
| Non-Hispanic Other | 2 (6.5) | 0 (0.0) | |
| Unknown | 1 (3.2) | 1 (5.3) | |
| Etiology of cirrhosis, n (%) | | | 0.48 |
| Alcohol | 25 (80.6) | 12 (63.2) | |
| MASH | 6 (19.4) | 4 (21.1) | |
| Viral | 1 (3.2) | 1 (5.3) | |
| Other | 3 (9.7) | 5 (26.3) | |
| MELD, median (IQR) | 30 (25.5-36) | 30 (24-36.5) | 0.83 |
| MELD-Na, median (IQR) | 33.1 (29-36.6) | 31.5 (27-37.3) | 0.62 |
| Baseline serum creatinine, median (mg/dL) | 2.6 (2.3-3.5) | 2.6 (2.1-2.8) | 0.48 |
| Baseline total bilirubin, median (mg/dL) | 6.5 (2.1-29.5) | 8.1 (2.1-27.3) | 0.95 |
| Baseline INR, median (IQR) | 1.9 (1.7-2.6) | 2.3 (1.8-2.9) | 0.15 |
| Baseline sodium, median (mEq/L) | 130 (126-133) | 132 (128.5-135.5) | 0.26 |
| AKI, n (%) | | | 0.10 |
| Stage 1 | 8 (25.8) | 10 (52.6) | |
| Stage 2 | 7 (22.6) | 4 (21.1) | |
| Stage 3 | 15 (48.4) | 4 (21.1) | |
| Baseline MAP, median (mmHg) (IQR) | 77.5 (75-80.2) | 79 (77-86.2) | 0.16 |
| Child-Pugh class, n (%) | | | 1.00 |
| A | 0 (0.0) | 0 (0.0) | |
| B | 8 (25.8) | 5 (26.3) | |
| C | 23 (74.2) | 14 (73.7) | |
| **ACLF, n (%)** | | | |
| Grade 1 | 6 (19.4) | 6 (31.6) | 0.60 |
| Grade 2 | 17 (54.8) | 8 (42.1) | |
| Grade 3 | 8 (25.8) | 5 (26.3) | |
| Initial Midodrine, n (%) | 24 (77.4) | 15 (78.9) | 1.00 |
| Initial Octreotide, n (%) | 22 (71.0) | 15 (78.9) | 0.74 |
| Prior NE or Terlipressin, n (%) | 4 (12.9) | 2 (10.5) | 1.00 |

**Supplementary Table 2. Primary and secondary outcomes between patients that tolerated terlipressin or NE**

| Outcome | Terlipressin (n=31) | Norepinephrine (n=45) | p-value |
|---|---|---|---|
| Verified Reversal of HRS, n (%) | 8 (25.8) | 7 (15.6) | 0.38 |
| AKI Response, n (%) | | | 0.13 |
| Full Response | 10 (32.3) | 6 (13.3) | |
| Partial Response | 4 (12.9) | 9 (20.0) | |
| No Response | 17 (54.8) | 30 (66.7) | |
| Overall Response | 14 (45.2) | 15 (33.3) | 0.34 |
| Median Days of Treatment (IQR) | 5 (4-8.5) | 5 (3-8) | 0.82 |
| Median MAP Pre-Treatment (mmHg) (IQR) | 77.5 (75-80.3) | 74 (71-83) | 0.26 |
| Median MAP 24h Post (mmHg) (IQR) | 79.5 (75-83) | 80.5 (73.5-86.5) | 0.72 |
| Median Change in MAP (mmHg) (IQR) | 1 (-3 to 6) [p=0.37] | 3 (-0.5-8.5) [p=0.003] | 0.26 |
| 90-day LT Rate, n (%) | 11 (35.5) | 18 (40.0) | 0.81 |
| **Overall Survival (OS), n (%)** | | | |
| 14-day | 23 (74.2) | 34 (75.6) | 1.00 |
| 30-day | 23 (74.2) | 31 (68.9) | 0.80 |
| 90-day | 22 (71) | 26 (57.8) | 0.33 |
| **Liver-Transplant Free Survival (LTFS), n (%)** | | | |
| 14-day | 20 (64.5) | 27 (60.0) | 0.81 |
| 30-day | 15 (48.4) | 18 (40.0) | 0.49 |
| 90-day | 11 (35.5) | 8 (17.8) | 0.11 |

**Supplementary Table 3. Adjusted sensitivity analyses for clinical outcomes in the full ITT cohort (n=100)**

| Outcome | Model | Covariate | Adjusted Estimate (95% CI) | p-value |
|---|---|---|---|---|
| Verified HRS Reversal | Logistic regression | Terlipressin (vs Norepinephrine) | 1.31 (0.41-4.40) | 0.65 |
| | | Male sex (vs Female) | 0.68 (0.20-2.26) | 0.52 |
| | | MELD-Na (per 1-unit increase) | 0.92 (0.83-1.01) | 0.11 |
| 90-day Overall Survival | Cox proportional hazards | Terlipressin (vs Norepinephrine) | 0.83 (0.43-1.60) | 0.58 |
| | | Male sex (vs Female) | 1.62 (0.79-3.33) | 0.19 |
| | | MELD-Na (per 1-unit increase) | 0.98 (0.93-1.04) | 0.55 |
| 90-day Liver Transplant-Free Survival | Cox proportional hazards (stratified*) | Terlipressin (vs Norepinephrine) | 0.78 (0.48-1.25) | 0.29 |
| | | Male sex (vs Female) | 1.48 (0.87-2.52) | 0.15 |

**Stratified by MELD-Na tertile (<=30.3, 30.3-37.0, >37.0) due to violation of the proportional hazards assumption, thus no HR estimated.*
