# Supplementary material for: Nonintensive Care Unit Norepinephrine as an Alternative to Terlipressin in Hepatorenal Syndrome–Acute Kidney Injury
Source: Gastro Hep Adv. 2026 Aug 12;5(11):101096. doi: 10.1016/j.gastha.2026.101096 (PMC13584103; doi:10.1016/j.gastha.2026.101096)
Supplement: Supplementary file 2 [file mmc2.pdf]

Gastro Hep Advances 2026;5:101096

ORIGINAL RESEARCH—CLINICAL

Nonintensive Care Unit Norepinephrine as an Alternative to Terlipressin in Hepatorenal Syndrome–Acute Kidney Injury

Vignesh Rajasekaran,[1] Xingxing S. Cheng,[2] Ethan Akama-Garren,[3] Juan Garcia,[4] Jason Phillips,[4] Kitty Deng,[4] Uerica Wang,[4] Elsa Sola,[3] Sajid Jalil,[3] Paul Kwo,[3] and Allison J. Kwong[3]

[1]*Division of Internal Medicine, Stanford University, Stanford, California;* [2]*Division of Nephrology, Stanford University, Stanford, California;* [3]*Division of Gastroenterology and Hepatology, Stanford University, Stanford, California; and* [4]*Department of Pharmacy, Stanford Health Care, Stanford, California*

BACKGROUND AND AIMS: Terlipressin is approved in the United States for the treatment of hepatorenal syndrome–acute kidney injury (HRS-AKI). At our institution, low-dose norepinephrine (NE) can be administered outside the intensive care unit (ICU) on a step-down unit for treatment of HRS-AKI. **METHODS:** We conducted a retrospective, single-center study of adults outside the ICU with HRS-AKI treated with terlipressin or NE from 2021 to 2025 at our institution. The primary outcome was verified reversal of HRS, defined as 2 consecutive serum creatinine measurements ≤1.5 mg/dL by day 14 and survival free of renal replacement therapy at least 10 days after treatment. Secondary outcomes included adverse events (AEs), AKI response, overall survival, and liver transplant–free survival. **RESULTS:** Among 50 patients treated within each cohort, baseline characteristics were generally balanced, except a higher proportion of males in the terlipressin group (76% vs 48%, $P = .007$). Verified HRS reversal (16% vs 14% $P = 1.00$), 90-day overall survival (64% vs 60%, log-rank $P = .83$), and 90-day liver transplant–free survival (30% vs 18%, log-rank $P = .47$) were comparable. Terlipressin was associated with higher discontinuation rates (38% vs 10%, $P = .002$) primarily from respiratory failure, peripheral ischemia, and gastrointestinal AEs. Median daily albumin was higher in the terlipressin group (25.0 vs 13.2 g/day, $P = .002$) but did not differ within the terlipressin cohort between patients who developed respiratory failure and those who did not. **CONCLUSION:** In this real-world cohort, terlipressin and non-ICU NE demonstrated comparable efficacy in HRS-AKI, but terlipressin was associated with higher rates of AEs and discontinuation. In centers where NE can be administered outside the ICU, NE remains a safe and cost-effective alternative to terlipressin.

Keywords: Hepatorenal Syndrome; HRS-AKI; Terlipressin; Norepinephrine; Cirrhosis; Acute Kidney Injury

Introduction

Hepatorenal syndrome (HRS) is a type of kidney injury typically seen in patients with advanced cirrhosis with ascites, recently reclassified into HRS-acute kidney injury (AKI) or HRS-chronic kidney disease.[1,2] It is thought to result from splanchnic vasodilation driven by an increased inflammatory state found in portal hypertension, followed by compensatory renal vasoconstriction and decreased renal perfusion.[3]

Current guidelines recommend vasoconstrictors, preferably terlipressin, as first-line in HRS-AKI.[4] Terlipressin is a synthetic vasopressin analog with increased selectivity to V1a receptors in the splanchnic circulation, effectively reducing portal inflow and increasing effective arterial blood volume. Although widely used in Europe for decades based on the REVERSE and OT-0401 trials, it was not approved by the Food and Drug Administration (FDA) for use in the United States until 2022 after the CONFIRM trial demonstrated improved verified HRS reversal vs placebo in HRS-AKI.[4–8]

Prior to terlipressin's approval, norepinephrine (NE) was the vasoconstrictor of choice for HRS-AKI in the United States. A key argument driving terlipressin adoption has been its ability to be administered outside the intensive care unit (ICU) without central venous access—an advantage that assumes NE requires ICU-level monitoring. Our institution has addressed this directly through a validated protocol for non-ICU NE administration in HRS-AKI on a step-down unit, operational since 2018.[9] Prior direct comparisons of terlipressin and NE have been limited to small, open-label trials conducted predominantly outside the United States, which generally showed comparable efficacy but were not designed to evaluate real-world safety signals or the specific context of institutionalized non-ICU

Abbreviations used in this paper: ACLF, acute-on-chronic liver failure; AE, adverse event; AKI, acute kidney injury; CI, confidence interval; FDA, Food and Drug Administration; HRS, hepatorenal syndrome; HRS-AKI, hepatorenal syndrome–acute kidney injury; ICA, International Club of Ascites; ICU, intensive care unit; LTFS, liver transplant–free survival; MAP, mean arterial pressure; MELD, Model for End-Stage Liver Disease; MELD-Na, Model for End-Stage Liver Disease–Sodium; NE, norepinephrine; OS, overall survival; RRT, renal replacement therapy.

Most current article

© 2026 The Authors. Published by Elsevier Inc. on behalf of American Gastroenterological Association Institute. This is an open access article under the CC BY license (http://creativecommons.org/licenses/by/4.0/).
2772-5723
https://doi.org/10.1016/j.gastha.2026.101096

NE delivery.[10–14] To our knowledge, no US study has evaluated terlipressin against an established non-ICU NE protocol in the post-FDA-approval era. Here, we aim to assess real-world efficacy and safety outcomes between non-ICU terlipressin and NE in HRS-AKI.

Materials and Methods

Ethical Considerations

The non-ICU NE protocol was developed as a quality improvement initiative and approved by the institutional Pharmacy and Therapeutics committee in 2018; institutional review board approval and informed consent were not required for the clinical administration of NE or terlipressin. The retrospective review of patient outcomes was reviewed and approved by the institutional review board (protocol #48531).

Cohort Definition

Adults (≥18 years) hospitalized at Stanford Healthcare from January 2021 to October 2025 with HRS-AKI who received terlipressin or NE were identified using diagnostic codes and medication administration records. A target sample size of 50 consecutive patients per vasoconstrictor was prespecified to allow balanced comparison. The study time frame was defined to encompass the period of institutional terlipressin availability while allowing a sufficient retrospective look back to capture a comparable volume of NE cases. HRS-AKI was defined according to the 2015 guidelines from the International Club of Ascites (ICA), which included (1) a diagnosis of cirrhosis and ascites, (2) a diagnosis of AKI according to ICA-AKI criteria, (3) no response after 2 consecutive days of diuretic withdrawal and plasma volume expansion with albumin 1 g/kg of body weight, (4) absence of shock, (5) no current or recent use of nephrotoxins, and (6) no macroscopic signs of structural kidney injury.[8] Additionally, patients in the ICU at the initiation of HRS-AKI treatment were excluded.

Baseline creatinine was determined as the lowest creatinine value within 3 months of admission; if unavailable, the search was extended to 6 months; otherwise, the admission value was used. AKI was defined as an increase in serum creatinine ≥0.3 mg/dL within 48 hours or >50% from baseline presumed to occur within the prior 7 days.[8] Stage 1 AKI was defined as an increase in serum creatinine ≥0.3 mg/dL or 1.5–2-fold from baseline; stage 2 AKI was an increase 2 to 3 fold from baseline; and stage 3 AKI was an increase >3 fold from baseline or serum creatinine >4.0 mg/dL with an acute increase >0.3 mg/dL or initiation of renal replacement therapy (RRT).[8] Proteinuria was permitted provided it did not exceed a threshold of 500 mg/day.

Norepinephrine was approved for the treatment of HRS-AKI outside of the ICU at our institution in May 2018, and terlipressin was available on the formulary starting in May 2023. After terlipressin became available, treatment selection was per clinician preference. However, terlipressin was avoided if patients had a serum creatinine >5 mg/dL, respiratory failure (SpO2 < 90%), or known pulmonary edema as per the package insert.[10] Midodrine and octreotide were discontinued prior to initiation of terlipressin or NE. Patients could cross over to the alternative vasoconstrictor if they did not tolerate or respond to the initial therapy. Institutional protocols for terlipressin and NE for HRS-AKI are described below.

Terlipressin Protocol

Patients in the terlipressin group received terlipressin 0.85 mg intravenously every 6 hours. Renal function was reassessed on day 4: terlipressin was discontinued if no improvement in serum creatinine occurred, continued at the same dose if creatinine decreased >30% from baseline, or increased to 1.7 mg every 6 hours if creatinine improved but decreased <30% from baseline per the package insert.[10] Treatment continued until serum creatinine fell below 1.5 mg/dL for 2 consecutive days or for a maximum of 14 days. Terlipressin was discontinued for oxygen saturation <90% on room air, failure to improve serum creatinine after 72 hours, return of creatinine to baseline, initiation of RRT, liver transplantation, transjugular intrahepatic portosystemic shunt placement, or initiation of vasopressors for treatment of shock. Signs of ischemia (chest pain, abdominal pain, diarrhea, and peripheral ischemia) prompted immediate discontinuation. Albumin was administered at 1 g/kg (maximum 100 g) on day 1, followed by 20–40 g daily on days 2–3, per the CONFIRM trial.[5] A modification to the protocol in 2023 allowed for albumin to be held in patients at high risk for development of pulmonary edema or heart failure, and dose escalation of terlipressin was avoided in patients with coronary artery disease, pulmonary edema, circulatory overload, or bronchospasm.

Norepinephrine Protocol

The NE protocol followed our institutional standard, similar to previously described.[9] Patients in the NE group received a continuous intravenous infusion via central venous catheter, initiated at 0.05 mcg/kg/min. The dose of NE was titrated in 0.03 mcg/kg/min increments no more frequently than every 4 hours to achieve a mean arterial pressure (MAP) increase of 10 mmHg above baseline, with a maximum dose of 0.14 mcg/kg/min. A MAP increase of ≥10 mmHg is consistent with prior protocols for NE in HRS and has been associated with treatment response.[9,11] All patients were to receive albumin 25 g daily. Treatment continued until serum creatinine recovered to less than 1.5 mg/dL or was discontinued if no reduction in creatinine occurred after 48 hours. Patients were ineligible for NE if they had a history of coronary artery disease, cardiomyopathy, or established arrhythmia, or if they met 2 or more systemic inflammatory response syndrome criteria.

Outcomes

The primary outcome was verified reversal of HRS, defined as 2 consecutive serum creatinine measurements ≤1.5 mg/dL obtained at least 2 hours apart by day 14, with survival free of RRT for at least 10 days following treatment completion as per the CONFIRM trial.[5] Secondary outcomes included response in kidney function, categorized as full response (improvement of AKI stage with return to within 0.3 mg/dL of baseline creatinine), partial response (improvement of AKI stage with serum creatinine remaining ≥0.3 mg/dL above baseline), or no response. Overall response was defined as achieving either a

Table 1. Baseline Characteristics of All Patients, Stratified by Terlipressin and Norepinephrine

Characteristic	Terlipressin (n = 50)	Norepinephrine (n = 50)	*P* value
Median age, y (IQR)	52.5 (40.8–61.8)	56.5 (44.2–63.8)	.52
Male sex, n (%)	38 (76.0)	24 (48.0)	.007
Race/ethnicity, n (%)			.78
Hispanic/Latino	21 (42.0)	21 (42.0)	
Non-Hispanic White	19 (38.0)	19 (38.0)	
Non-Hispanic Black	1 (2.0)	2 (4.0)	
Non-Hispanic Asian	5 (10.0)	4 (8.0)	
Non-Hispanic other	2 (4.0)	4 (8.0)	
Unknown	2 (4.0)	0 (0.0)	
Etiology of cirrhosis, n (%)			.87
Alcohol	37 (74.0)	37 (74.0)	
MASH	10 (20.0)	8 (16.0)	
Viral	2 (4.0)	4 (8.0)	
Other	8 (16.0)	8 (16.0)	
MELD, median (IQR)	30 (24–37)	31 (27–37)	.44
MELD-Na, median (IQR)	32 (29–37)	33 (29–37)	.74
Baseline serum creatinine, median (mg/dL)	2.6 (2.3–3.5)	2.7 (2.3–3.7)	.43
Baseline total bilirubin, median (mg/dL)	7.3 (2–29.6)	5.9 (2.3–21.3)	.79
Baseline INR, median (IQR)	2.2 (1.7–2.7)	2.1 (1.9–2.7)	.77
Baseline sodium, median (mEq/L)	130 (126–135)	133 (129–137)	.13
Daily albumin (g) on therapy, median (IQR)	25.0 (12.5–41.7)	13.2 (5.3–24.0)	.002
AKI, n (%)			.26
Stage 1	20 (40.0)	12 (24.0)	
Stage 2	11 (22.0)	9 (18.0)	
Stage 3	19 (38.0)	27 (54.0)	
Baseline MAP, median (mm Hg) (IQR)	78 (76–82)	75 (71–83)	.10
Child-Pugh class, n (%)			1.00
A	0 (0.0)	0 (0.0)	
B	13 (26.0)	12 (24.0)	
C	37 (74.0)	38 (76.0)	
ACLF, n (%)			.62
Grade 1	12 (24.0)	15 (30.0)	
Grade 2	25 (50.0)	20 (40.0)	
Grade 3	13 (26.0)	15 (30.0)	
Initial midodrine, n (%)	39 (78.0)	45 (90.0)	.17
Initial octreotide, n (%)	37 (74.0)	45 (90.0)	.07
Prior NE or terlipressin, n (%)	6 (12.0)	7 (14.0)	1.00

INR, international normalized ratio; IQR, interquartile range; MASH, metabolic dysfunction-associated steatohepatitis.

full or partial response. Additional secondary outcomes included achievement of MAP goal (increase ≥10 mmHg from baseline), overall survival (OS) at 14, 30, and 90 days, liver transplant–free survival (LTFS) at 14, 30, and 90 days, adverse events (AEs), and treatment discontinuation. Adverse events were categorized as arrhythmia, cyanosis, gastrointestinal symptoms, respiratory failure, peripheral ischemia, or other.

Statistical Analysis

Baseline characteristics were compared between the terlipressin and NE groups. Continuous variables were reported as median with interquartile range and compared using the Wilcoxon rank-sum test. Within-group changes in MAP from baseline to 24 hours post-treatment were assessed using the paired Wilcoxon signed-rank test. Categorical variables were reported as frequency with percentage and compared between treatment groups using Fisher's exact test, the chi-square test, or the one-way analysis of variance, as appropriate. To address the baseline sex imbalance and other potential confounders, prespecified adjusted sensitivity analyses were performed. Verified HRS reversal was modeled using multivariable logistic regression; 90-day OS and LTFS were modeled using Cox proportional hazards regression, each adjusted for treatment, sex, and Model for End-Stage Liver Disease–Sodium (MELD-Na). The proportional hazards assumption was assessed using Schoenfeld residuals; MELD-Na violated this assumption in the LTFS model ($P < .05$) and was entered as a stratification variable using cohort-derived tertiles (≤30.3, 30.3–37.0, >37.0).

OS and LTFS were estimated using the Kaplan-Meier method, with time zero defined as the date of treatment initiation. Patients were followed for 90 days with complete follow-up. For OS, patients were censored at 90 days if death did not occur. For LTFS, the primary endpoint was defined as the composite of death or liver transplantation; patients who remained alive and transplant free were censored at 90 days. Differences in survival curves between treatment groups were

Table 2. Primary and Secondary Outcomes, Stratified by Terlipressin and Norepinephrine

Outcome	Terlipressin (n = 50)	Norepinephrine (n = 50)	*P* value
Verified reversal of HRS, n (%)	8 (16.0)	7 (14.0)	1.00
AKI response, n (%)			.45
Full response	10 (20.0)	6 (12.0)	
Partial response	6 (12.0)	9 (18.0)	
No response	34 (68.0)	35 (70.0)	
Overall response	16 (32.0)	15 (30.0)	1.00
Median days of treatment (IQR)	3 (1–5)	4 (2–7)	.012
Median MAP pre-treatment (mmHg) (IQR)	78.2 (75.5–82.0)	75.0 (71.3–83.0)	.10
Median MAP 24 h post-treatment (mmHg) (IQR)	80.5 (76.5–85.0)	80.5 (73.5–86.4)	.85
Median change in MAP (mmHg) (IQR)	0.5 (−3.1 to 6.8) [*P*> = .45]	3.0 (−0.4 to 8.5) [*P* = .001]	.13
Liver transplant within 90 d, n (%)	18 (36.0)	21 (42.0)	.68
Overall survival (OS), n (%)			
14-d	36 (72.0)	38 (76.0)	.82
30-d	34 (68.0)	35 (70.0)	1.00
90-d	32 (64.0)	30 (60.0)	.84
Liver transplant–free survival (LTFS), n (%)			
14-d	29 (58.0)	30 (60.0)	1.00
30-d	19 (38.0)	21 (42.0)	.84
90-d	15 (30.0)	9 (18.0)	.24

assessed using the log-rank test. A subgroup analysis of survival outcomes was performed among patients who completed treatment without premature discontinuation due to AEs.

A 2-tailed *P* value <.05 was considered statistically significant. All analyses were performed using R version 4.5.1 (R Foundation for Statistical Computing, Vienna, Austria).

Results

Demographics

During the study period, 50 adult patients received terlipressin and 50 patients received NE (Table 1). The treatment groups were well-balanced with respect to age, race, and ethnicity. Alcohol-associated liver disease was the predominant etiology of cirrhosis in both groups, with no significant differences observed between cohorts. Baseline disease severity and clinical characteristics—including MELD, MELD-Na, AKI stage, acute-on-chronic liver failure (ACLF) grade, and median MAP—were also similar at the time of treatment initiation. Overall, the median MELD was 30 (interquartile range 26–37), and 28% had ACLF grade 3. The only significant demographic difference observed was biological sex; the terlipressin cohort had a significantly higher proportion of male patients compared to the NE cohort (76%–48%, *P* = .007).

Efficacy

In the intention-to-treat analysis, 16% of patients receiving terlipressin reached the primary endpoint of HRS reversal vs 14% of patients receiving NE (*P* = 1.00). There were no statistically significant differences between the terlipressin and NE cohorts regarding the rates of categorical AKI response (Table 2). At 24 hours post-initiation, patients treated with NE experienced a significant increase in MAP from baseline (*P* = .001), whereas those treated with terlipressin did not.

OS and LTFS were similar between groups over the 90-day follow-up, with no significant differences at 14, 30, or 90 days. At 90 days, Kaplan–Meier analysis showed comparable OS between the terlipressin and NE groups (64% vs 60%, log-rank *P* = .83), as well as LTFS (30% vs 18%, log-rank *P* = .47) (Figure). Listing status at therapy initiation (12% vs 14%, *P* = 1.00) and transplantation rates (36% vs 42%, *P* = .68) were comparable between groups.

After adjustment for sex and MELD-Na, there were no significant differences between treatment groups for verified HRS reversal (adjusted odds ratio: 1.31, 95% confidence interval [CI]: 0.41–4.40), 90-day OS (adjusted hazard ratio: 0.83; 95% CI: 0.43–1.60), or 90-day LTFS (adjusted hazard ratio: 0.78, 95% CI: 0.48–1.25) (Supplementary Table 3).

Adverse Events and Discontinuation

Safety outcomes and AEs are summarized in Table 3. Patients treated with terlipressin experienced a significantly higher burden of toxicity compared to those treated with NE. The rate of treatment discontinuation due to AEs was significantly higher in the terlipressin cohort (38.0% vs 10.0%, *P* = .002), predominantly driven by respiratory and ischemic complications. The incidence of respiratory failure, peripheral ischemia, and gastrointestinal symptoms was exclusively observed in the terlipressin arm (16.0%, 12.0%, and 10.0%, respectively). No significant difference was observed in the rates of arrhythmia (10.0% vs 12.0%, *P* = 1.00) or other AEs between the 2 groups.

The median daily albumin received during therapy was significantly higher in the terlipressin group compared to the NE group (25.0 vs 13.2 g/day; *P* = .002); however, within

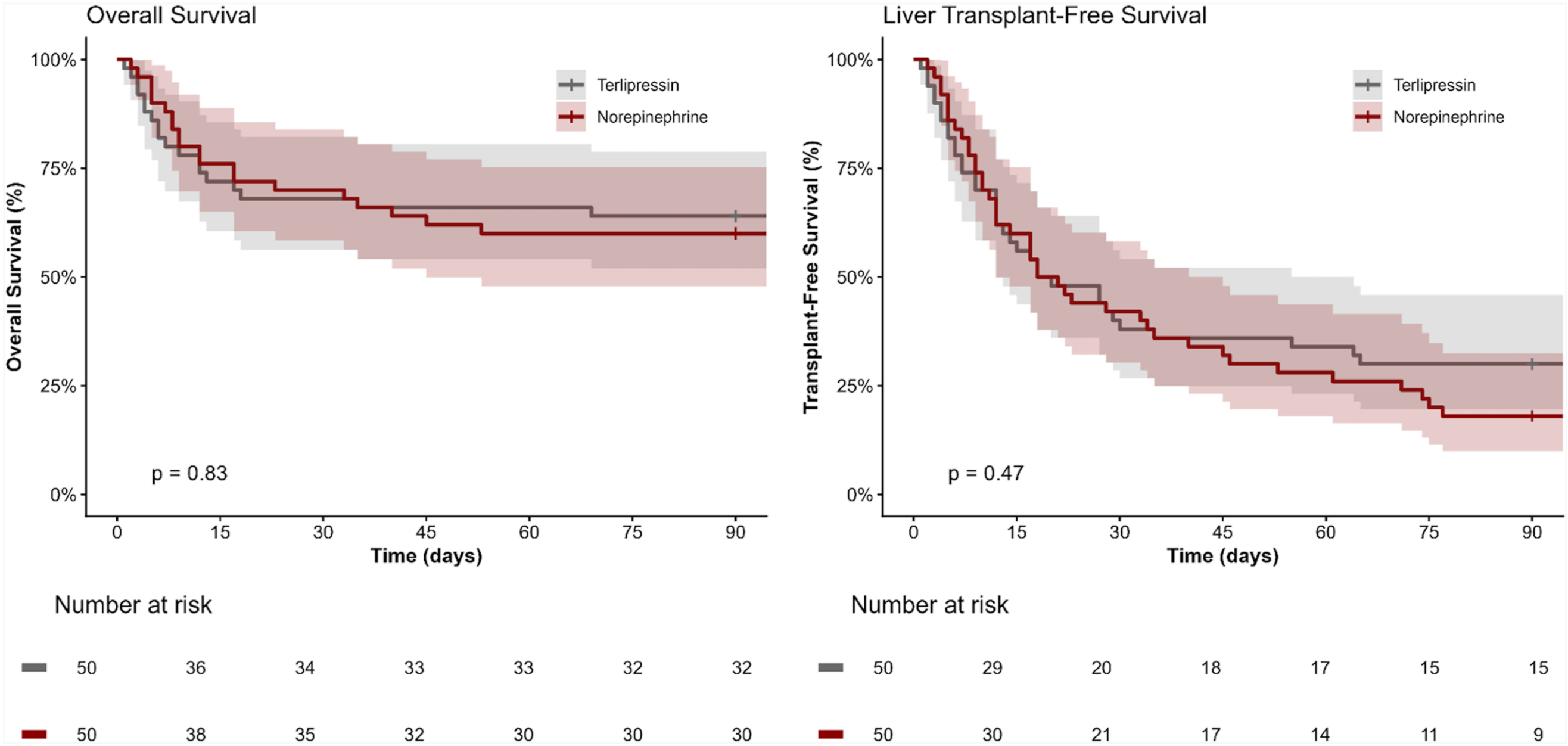

Figure. Kaplan–Meier estimates of overall survival and liver transplant–free survival over 90 days of follow-up, comparing terlipressin (gray) and norepinephrine (red). Shaded regions represent 95% confidence intervals. The number of patients at risk in each group is shown below each panel. Time zero was defined as the date of vasoconstrictor initiation. Between-group differences were assessed using the log-rank test (overall survival, $P = .83$; liver transplant–free survival, $P = .47$).

the terlipressin cohort, was comparable between patients who experienced respiratory failure and those who did not (19.4 vs 25.0 g/day; $P = .45$). There were no other significant differences in baseline characteristics between patients who tolerated terlipressin (n = 31) vs those that did not (n = 19), including ACLF grade (Supplementary Table 1).

Given the higher rate of AE-related discontinuation in the terlipressin arm, a post hoc analysis was also performed to evaluate efficacy specifically among patients that did not discontinue due to AEs (terlipressin n = 31 vs NE n = 45). In this subgroup analysis, there were no significant differences seen in outcomes for HRS reversal, OS, or LTFS (Supplementary Table 2). Although the median duration of treatment was shorter in the terlipressin arm (3 [1–5] vs 4 [2–7] days, $P = .012$; Table 2), this appears attributable to AE–related discontinuation rather than early response, as the difference resolved among patients who did not discontinue (5 [4–8.5] vs 5 [3–8] days, $P = .82$; Supplementary Table 2).

Discussion

In this real-world, retrospective cohort analysis, terlipressin and NE demonstrated comparable clinical efficacy in HRS-AKI; however, significantly higher rates of AEs and treatment discontinuation were observed in the terlipressin group. To our knowledge, this is the first US head-to-head comparison of these 2 vasoconstrictors in a real-world cohort following FDA approval of terlipressin in 2022 and the first to evaluate terlipressin against an institutionally established protocol for non-ICU NE administration.

Prior US clinical practice for HRS-AKI has been constrained by the perceived need for ICU-level monitoring of NE, which has driven adoption of midodrine-octreotide as a first-line alternative and contributed to the regulatory and clinical case for terlipressin. This practice pattern is reflected in a recent national survey, in which only 12% of US centers reported the ability to administer low-dose NE outside the ICU for HRS-AKI.[12] The present analysis

Table 3. Adverse Events and Discontinuation Rate, Stratified by Terlipressin and Norepinephrine

AE type, n (%)	Terlipressin (n = 50)	Norepinephrine (n = 50)	*P* value
Arrhythmia	5 (10.0)	6 (12.0)	1.00
Cyanosis	1 (2.0)	0 (0.0)	1.00
Gastrointestinal symptoms	5 (10.0)	0 (0.0)	.056
Respiratory failure	8 (16.0)	0 (0.0)	.006
Peripheral ischemia	6 (12.0)	0 (0.0)	.027
Other	1 (2.0)	3 (6.0)	.62
Discontinuation, n (%)	19 (38.0)	5 (10.0)	.002

demonstrates that this protocol for non-ICU NE produces clinical outcomes comparable to terlipressin in a real-world post-FDA-approval era, with a more favorable safety profile in this cohort.

While several randomized trials have directly compared terlipressin and NE in HRS-AKI, these studies have largely been open-label, conducted outside the United States, and limited by sample size.[11,13–15] They generally showed comparable efficacy but often failed to capture the safety signals now observed in larger analyses. Terlipressin has shown superiority over NE in an ACLF-restricted population; however, this trial's narrower ACLF-selected population and infusion-based protocol may account for the differing efficacy signal.[16] A recent meta-analysis showed a discontinuation rate of 5.3% for terlipressin vs 2.7% for NE, whereas the CONFIRM trial reported a discontinuation rate as high as 12%.[5,17] The HRS-HARMONY consortium recently reported the largest US multicenter experience with terlipressin post-FDA approval, with an overall discontinuation rate of approximately 17% due to any AEs, with 9.5% from hypoxia.[18] Notably, the 16% incidence of respiratory failure in our terlipressin arm mirrors the 20% rate recently reported by Ma et al,[19] in a large international prospective real-world study of terlipressin in HRS-AKI. Despite similar protocol recommendations to FDA-labeled dosing and discontinuation criteria, single-center practice patterns and patient-level heterogeneity likely contribute to variability in observed AE-driven discontinuation rates across real-world cohorts. The qualitative pattern of meaningful AE-driven discontinuation with terlipressin exceeding clinical trial benchmarks is concordant across our cohort, HRS-HARMONY, and the international Ma et al, cohort, supporting the reproducibility of real-world terlipressin safety concerns.

A critical factor driving this respiratory toxicity may be the cumulative oncotic load. Current guidelines for HRS-AKI recommend albumin 20 to 40 g daily; however, there is limited evidence defining the ideal dose and duration of therapy.[20] The most recent joint Acute Disease Quality Initiative and ICA guidelines on HRS caution against excessive albumin in patients who are not clinically intravascularly depleted.[21] While both institutional protocols recommended use of albumin while on vasoconstrictor therapy, we observed a higher median daily albumin load in the terlipressin cohort than in the NE arm, though still within guideline recommendations. This difference likely reflects protocol-driven dosing: the terlipressin protocol recommended albumin 1 g/kg on day 1 followed by 20 to 40 g daily per FDA labeling, whereas the NE protocol specified a fixed 25 g daily. The front-loaded albumin dosing, combined with terlipressin's intrinsic effects on pulmonary vasculature, may have contributed to the incidence of hypoxia and volume overload. However, within the terlipressin group itself, there was no significant difference in median daily albumin dose between patients who developed respiratory failure and those who did not, which aligns with findings from HRS-HARMONY and Ma et al.[18,19] The lack of correlation suggests that terlipressin-induced respiratory failure may occur even with judicious albumin administration and may not be entirely attributable to volume overload.

The pharmacokinetic differences between the 2 agents—intermittent bolus dosing with prolonged duration of action vs continuous infusion—could also theoretically result in different patterns of renal perfusion. Continuous terlipressin infusion, which was not available at our center during the study period, has shown a superior safety profile primarily in the setting of acute variceal bleeding, with some evidence suggesting it may similarly improve tolerability in HRS-AKI by minimizing total cumulative exposure and peak-dose toxicity.[22,23] However, the HRS-HARMONY and Ma et al data suggest that respiratory AE rates do not differ substantially between bolus and infusion administration in a real-world population, lowering expectations that continuous terlipressin infusion alone will resolve the respiratory AE signal.

The HRS-HARMONY findings additionally underscore important features of real-world terlipressin use that strengthen the rationale for non-ICU NE as an alternative. Notably, 37.9% of HRS-HARMONY patients required subsequent NE therapy for hemodynamic support during the same admission, and 23.6% received terlipressin within the ICU.[18] These data suggest that a substantial fraction of HRS-AKI patients ultimately require ICU-level vasopressor management regardless of initial vasoconstrictor choice.

The clinical and economic implications of this comparison are substantial. The daily acquisition cost of terlipressin is at least $4,000, compared with negligible drug costs for generic NE.[24] When NE can be administered outside the ICU under an established institutional protocol, avoiding ICU days further amplifies the cost advantage. In centers without comparable infrastructure for non-ICU vasoconstrictor administration, terlipressin's ability to be given without central venous access or ICU monitoring represents a meaningful clinical advantage.

These findings must be interpreted in the context of several limitations. First, the retrospective, single-center design introduces inherent selection bias and limits generalizability. Our institutional criteria avoiding terlipressin in patients with significant respiratory compromise or creatinine >5 mg/dL may have resulted in unmeasured confounding, with the NE cohort potentially enriched for patients with occult cardiopulmonary dysfunction or more severe disease. Partly mitigating this concern is the fact that baseline MELD-Na, AKI stage, and ACLF grade were well-balanced between groups, and adjusted analyses accounting for sex and MELD-Na yielded treatment effect estimates consistent with the unadjusted results across all 3 outcomes. Second, echocardiography was not systematically performed, and subclinical cirrhotic cardiomyopathy as a contributor to the observed ischemic and respiratory AEs therefore cannot be fully excluded. Third, the sample size limits the power of extensive subgroup analyses and increases the risk of type II error, though it represents one of the largest real-world cohorts comparing these 2 agents to date. Finally, the relatively preserved baseline MAP at vasoconstrictor

initiation in both arms reflects structural features of current US HRS-AKI diagnosis and management—patients met ICA 2015 criteria requiring documented non-response to albumin volume expansion prior to vasoconstrictor eligibility and our institutional practice guidelines recommending stepwise midodrine-octreotide prior to terlipressin or NE initiation.

Conclusion

Terlipressin represents a therapeutic option with a complex risk-benefit profile in HRS-AKI. While it offers the potential advantage of avoiding ICU admission at centers where NE would require ICU-level monitoring, it carries significant risks of respiratory and peripheral ischemic toxicity. Clinical implementation should focus on rigorous patient selection, more conservative albumin dosing to avoid those at high risk of respiratory failure, and potentially earlier vasoconstrictor initiation. Norepinephrine, when administered under an established non-ICU institutional protocol, remains a viable and cost-effective alternative for the treatment of HRS-AKI.

Declaration of Generative AI and AI-Assisted Technologies in the Writing Process

AI tools (Claude, Anthropic) were used to assist with code development and language editing; all analyses, interpretations, and conclusions were performed and verified by the authors.

Supplementary Materials

Material associated with this article can be found, in the online version, at https://doi.org/10.1016/j.gastha.2026.101096.

References

1. Gupta K, Burwhal A, Law C, et al. Acute kidney injury and hepatorenal syndrome in cirrhosis. World J Gastroenterol 2021;27:3984–4003.
2. Pose E, Piano S, Juanola A, et al. Hepatorenal syndrome in cirrhosis. Gastroenterology 2024;166:588–604.e1.
3. Allegretti AS, Patidar KR, Ma AT, et al. From past to present to future: terlipressin and hepatorenal syndrome-acute kidney injury. Hepatology 2025;81:1878–1897.
4. Garcia-Tsao G, Abraldes JG, Rich NE, et al. AGA clinical practice update on the use of vasoactive drugs and intravenous albumin in cirrhosis: expert review. Gastroenterology 2024;166:202–210.
5. Wong F, Pappas S, Curry M, et al. Terlipressin plus albumin for the treatment of type 1 hepatorenal syndrome. N Engl J Med 2021;384:818–828.
6. Sanyal AJ, Boyer T, Garcia-Tsao G, et al. A randomized, prospective, double-blind, placebo-controlled trial of terlipressin for type 1 hepatorenal syndrome. Gastroenterology 2008;134:1360–1368.
7. Boyer TD, Sanyal A, Wong F, et al. Terlipressin plus albumin is more effective than albumin alone in improving renal function in patients with cirrhosis and hepatorenal syndrome type 1. Gastroenterology 2016;150:1579–1589.e2.
8. Angeli P, Gines P, Wong F, et al. Diagnosis and management of acute kidney injury in patients with cirrhosis: revised consensus recommendations of the International Club of Ascites. Gut 2015;64:531–537.
9. Kwong A, Kim WR, Kwo PY, et al. Feasibility and effectiveness of norepinephrine outside the intensive care setting for treatment of hepatorenal syndrome. Liver Transpl 2021;27:1095–1105.
10. Mallinckrodt Pharmaceuticals. Terlivaz (Terlipressin) for injection prescribing information. 2022. https://www.accessdata.fda.gov/drugsatfda_docs/label/2022/022231s000lbl.pdf. Accessed June 1, 2026.
11. Singh V, Ghosh S, Singh B, et al. Noradrenaline vs. terlipressin in the treatment of hepatorenal syndrome: a randomized study. J Hepatol 2012;56:1293–1298.
12. Cullaro G, Im G, Chandna S, et al. The management of hepatorenal syndrome–acute kidney injury (HRS-AKI): a national survey of hepatology provider practices. Hepatol Commun 2026;10:e0905.
13. Alessandria C, Ottobrelli A, Debernardi-Venon W, et al. Noradrenalin vs terlipressin in patients with hepatorenal syndrome: a prospective, randomized, unblinded, pilot study. J Hepatol 2007;47:499–505.
14. Sharma P, Kumar A, Shrama BC, et al. An open label, pilot, randomized controlled trial of noradrenaline *versus* terlipressin in the treatment of type 1 hepatorenal syndrome and predictors of response. Am J Gastroenterol 2008;103:1689–1697.
15. Malik A, Malik MI, Qureshi S, et al. Efficacy and safety of terlipressin and albumin vs. noradrenaline and albumin in adult patients with hepatorenal syndrome: a systematic review and meta-analysis. Ann Hepatol 2024;29:101495.
16. Arora V, Maiwall R, Rajan V, et al. Terlipressin is superior to noradrenaline in the management of acute kidney injury in acute on chronic liver failure. Hepatology 2020;71:600–610.
17. Olson JC, Subramanian RM. Comparative efficacy of terlipressin and norepinephrine for treatment of hepatorenal syndrome-acute kidney injury: a systematic review and meta-analysis. PLoS One 2024;19:e0296690.
18. Patidar KR, Cullaro G, Orman ES, et al. U.S. experience with terlipressin in hepatorenal syndrome: Response, mortality, and transplant outcomes - results from the HRS harmony consortium. Clin Gastroenterol Hepatol 2026 May 8 [Epubahead of print].
19. Ma AT, Juanola A, Patidar K, et al. Real-world use of terlipressin in cirrhosis and acute kidney injury: frequent use beyond hepatorenal syndrome. Clin Gastroenterol Hepatol 2026;24:1079–1091.
20. Khemichian S, Nadim MK, Terrault NA. Update on hepatorenal syndrome: from pathophysiology to treatment. Annu Rev Med 2025;76:373–387.
21. Nadim MK, Kellum JA, Forni L, et al. Acute kidney injury in patients with cirrhosis: Acute Disease Quality Initiative (ADQI) and International Club of Ascites (ICA) joint multidisciplinary consensus meeting. J Hepatol 2024;81:163–183.

22. Liu D, Testro A, Majumdar A, et al. The current applications and future directions of terlipressin. Hepatol Commun 2025;9:e0685.
23. Hassan M, Merza N, Nawras Y, et al. Continuous vs. intermittent terlipressin infusion for portal hypertension: a systematic review and meta-analysis. Ann Med Surg 2023;85:5001–5010.
24. Huang X, Bindra J, Chopra I, et al. Treatment-related cost analysis of terlipressin for adults with hepatorenal syndrome with rapid reduction in kidney function. Adv Ther 2023;40:5432–5446.

Received June 1, 2026. Accepted August 6, 2026.

Correspondence:
Address correspondence to: Allison J. Kwong, MD, Division of Gastroenterology and Hepatology, Department of Medicine, Stanford University, 430 Broadway Street 3F, Redwood City, California 94063. e-mail: ajk@stanford.edu.

Authors' Contributions:
Vignesh Rajasekaran, Allison J. Kwong, and Xingxing S. Cheng: Concept and study design. Vignesh Rajasekaran, Ethan Akama-Garren, and Juan Garcia: Data acquisition and statistical analysis. Vignesh Rajasekaran and Allison J. Kwong: Manuscript drafting. Xingxing S. Cheng, Juan Garcia, Jason Phillips, Kitty Deng, Uerica Wang, Elsa Sola, Sajid Jalil, Paul Kwo, and Allison J. Kwong: Critical revision of the manuscript. Allison J. Kwong: Study supervision. All authors approved the final version of the manuscript.

Conflicts of Interest:
The authors disclose no conflicts.

Funding:
The authors report no funding.

Ethical Statement:
The non-ICU norepinephrine protocol was developed as a quality improvement initiative and approved by the institutional Pharmacy and Therapeutics committee in 2018; institutional review board approval and informed consent were not required for the clinical administration of norepinephrine or terlipressin. The retrospective review of patient outcomes was reviewed and approved by the institutional review board (protocol #48531).

Data Transparency Statement:
The data and analytic code that support the findings of this study are available from the corresponding author upon reasonable request. Individual patient-level data are not publicly available due to institutional privacy and data-governance restrictions.

Reporting Guidelines:
This study was conducted in accordance with the Strengthening the Reporting of Observational Studies in Epidemiology (STROBE) guidelines for cohort studies.

Gastro Hep Advances, Volume 5

Supplemental information

Nonintensive Care Unit Norepinephrine as an Alternative to Terlipressin in Hepatorenal Syndrome–Acute Kidney Injury

Vignesh Rajasekaran, Xingxing S. Cheng, Ethan Akama-Garren, Juan Garcia, Jason Phillips, Kitty Deng, Uerica Wang, Elsa Sola, Sajid Jalil, Paul Kwo, and Allison J. Kwong

Supplementary Table 1. Baseline characteristics of patients that tolerated terlipressin versus discontinued

Characteristic	Tolerated (n=31)	Discontinued (n=19)	p-value
Median age, years (IQR)	52 (42-58.5)	60 (39.5-70)	0.31
Male sex, n (%)	23 (74.2)	15 (78.9)	1.00
Race/Ethnicity, n (%)			0.74
Hispanic/Latino	14 (45.2)	7 (36.8)	
Non-Hispanic White	11 (35.5)	8 (42.1)	
Non-Hispanic Black	0 (0.0)	1 (5.3)	
Non-Hispanic Asian	3 (9.7)	2 (10.5)	
Non-Hispanic Other	2 (6.5)	0 (0.0)	
Unknown	1 (3.2)	1 (5.3)	
Etiology of cirrhosis, n (%)			0.48
Alcohol	25 (80.6)	12 (63.2)	
MASH	6 (19.4)	4 (21.1)	
Viral	1 (3.2)	1 (5.3)	
Other	3 (9.7)	5 (26.3)	
MELD, median (IQR)	30 (25.5-36)	30 (24-36.5)	0.83
MELD-Na, median (IQR)	33.1 (29-36.6)	31.5 (27-37.3)	0.62
Baseline serum creatinine, median (mg/dL)	2.6 (2.3-3.5)	2.6 (2.1-2.8)	0.48
Baseline total bilirubin, median (mg/dL)	6.5 (2.1-29.5)	8.1 (2.1-27.3)	0.95
Baseline INR, median (IQR)	1.9 (1.7-2.6)	2.3 (1.8-2.9)	0.15
Baseline sodium, median (mEq/L)	130 (126-133)	132 (128.5-135.5)	0.26
AKI, n (%)			0.10
Stage 1	8 (25.8)	10 (52.6)	
Stage 2	7 (22.6)	4 (21.1)	
Stage 3	15 (48.4)	4 (21.1)	
Baseline MAP, median (mmHg) (IQR)	77.5 (75-80.2)	79 (77-86.2)	0.16
Child-Pugh class, n (%)			1.00
A	0 (0.0)	0 (0.0)	
B	8 (25.8)	5 (26.3)	
C	23 (74.2)	14 (73.7)	
**ACLF, n (%)**			
Grade 1	6 (19.4)	6 (31.6)	0.60
Grade 2	17 (54.8)	8 (42.1)	
Grade 3	8 (25.8)	5 (26.3)	
Initial Midodrine, n (%)	24 (77.4)	15 (78.9)	1.00
Initial Octreotide, n (%)	22 (71.0)	15 (78.9)	0.74
Prior NE or Terlipressin, n (%)	4 (12.9)	2 (10.5)	1.00

Supplementary Table 2. Primary and secondary outcomes between patients that tolerated terlipressin or NE

Outcome	Terlipressin (n=31)	Norepinephrine (n=45)	p-value
Verified Reversal of HRS, n (%)	8 (25.8)	7 (15.6)	0.38
AKI Response, n (%)			0.13
Full Response	10 (32.3)	6 (13.3)	
Partial Response	4 (12.9)	9 (20.0)	
No Response	17 (54.8)	30 (66.7)	
Overall Response	14 (45.2)	15 (33.3)	0.34
Median Days of Treatment (IQR)	5 (4-8.5)	5 (3-8)	0.82
Median MAP Pre-Treatment (mmHg) (IQR)	77.5 (75-80.3)	74 (71-83)	0.26
Median MAP 24h Post (mmHg) (IQR)	79.5 (75-83)	80.5 (73.5-86.5)	0.72
Median Change in MAP (mmHg) (IQR)	1 (-3 to 6) [p=0.37]	3 (-0.5-8.5) [p=0.003]	0.26
90-day LT Rate, n (%)	11 (35.5)	18 (40.0)	0.81
**Overall Survival (OS), n (%)**			
14-day	23 (74.2)	34 (75.6)	1.00
30-day	23 (74.2)	31 (68.9)	0.80
90-day	22 (71)	26 (57.8)	0.33
**Liver-Transplant Free Survival (LTFS), n (%)**			
14-day	20 (64.5)	27 (60.0)	0.81
30-day	15 (48.4)	18 (40.0)	0.49
90-day	11 (35.5)	8 (17.8)	0.11

Supplementary Table 3. Adjusted sensitivity analyses for clinical outcomes in the full ITT cohort (n=100)

Outcome	Model	Covariate	Adjusted Estimate (95% CI)	p-value
Verified HRS Reversal	Logistic regression	Terlipressin (vs Norepinephrine)	1.31 (0.41-4.40)	0.65
		Male sex (vs Female)	0.68 (0.20-2.26)	0.52
		MELD-Na (per 1-unit increase)	0.92 (0.83-1.01)	0.11
90-day Overall Survival	Cox proportional hazards	Terlipressin (vs Norepinephrine)	0.83 (0.43-1.60)	0.58
		Male sex (vs Female)	1.62 (0.79-3.33)	0.19
		MELD-Na (per 1-unit increase)	0.98 (0.93-1.04)	0.55
90-day Liver Transplant-Free Survival	Cox proportional hazards (stratified*)	Terlipressin (vs Norepinephrine)	0.78 (0.48-1.25)	0.29
		Male sex (vs Female)	1.48 (0.87-2.52)	0.15

**Stratified by MELD-Na tertile (<=30.3, 30.3-37.0, >37.0) due to violation of the proportional hazards assumption, thus no HR estimated.*
